# Determinants of household adoption of clean energy with its rural–urban disparities in Bangladesh

**DOI:** 10.1038/s41598-024-52798-7

**Published:** 2024-01-29

**Authors:** Iqramul Haq, Maruf Khan, Sharanon Chakma, Md. Ismail Hossain, Shuvongkar Sarkar, Md. Rayhan Ali Rejvi, Md. Salauddin, Md Mizanur Rahman Sarker

**Affiliations:** 1 Department of Agricultural Statistics, Sher-e-Bangla Agricultural University, Dhaka, 1207 Bangladesh; 2 Department of Agricultural Economics, Sher-e-Bangla Agricultural University, Dhaka, 1207 Bangladesh; 3 Department of Statistics, Jagannath University, Dhaka, 1100 Bangladesh; 4 Department of Mathematics and Natural Sciences, BRAC University, Dhaka, 1212 Bangladesh; 5 Criminal Investigation Department, Dhaka, Bangladesh

**Keywords:** Environmental sciences, Environmental social sciences, Energy science and technology

## Abstract

This study aims to investigate factors influencing the adoption of clean energy among households in Bangladesh, using Blinder-Oaxaca decomposition and extended probit regression model with data from the 2019 Bangladesh multiple indicator cluster survey. Small households, primarily Muslim and urban dwellers, who speak the Bengali language and are Internet and mobile users, were likelier to adopt cleaner fuels than their counterparts. On the contrary, households residing in the Barisal, Khulna, Rajshahi, and Rangpur divisions, belonging to poor and middle-class households, with household heads aged 15–64 and without formal education, were less likely to adopt cleaner fuels than their counterparts. The concentration curve revealed socioeconomic inequality in the adoption of clean energy, particularly favouring richer households in urban and rural areas. Further analysis using the Blinder-Oaxaca decomposition showed that urban residents showed a higher probability of adopting clean energy, with a significant difference of 0.508 compared to rural areas. Regarding the endowment effect, poor wealth quintile contributed the most, followed by the ownership of rented dwellings and the middle wealth quintile. The Bengali differential effect made the largest contribution to this aspect of the disparity, followed by the exposure of the Internet and the influence of the Dhaka and Chattogram divisions. The detailed analysis provides valuable insights for policymakers and practitioners on the issue of disparities in the adoption of clean energy between urban and rural areas in Bangladesh.

## Introduction

The increasing global demand for energy, driven by urbanization and population growth, underscores the critical need to understand the essential role of coal-generated power in both the global electricity and environment impact^1^. Addressing the climate change, reducing electricity production costs, modernize infrastructure, and providing power to remote areas necessitate the three essential elements of decentralization, decarbonization, and democratization (the “three Ds”) in the global energy sector^2^. Since the ratification of the Paris Agreement, the achievement of carbon neutrality has assumed a more significant role on the world stage^3,4^

The achievement of a low-carbon economy is particularly important for developing countries because they depend on fossil fuels. Bangladesh is a developing nation on the Indian subcontinent that has not yet completely abandoned its dependence on fossil fuels for electricity generation^5^. Bangladesh has successfully evolved from being ridiculed as a "bottomless basket" to being a "role model" economy for other developing nations^6^. During the last 30 years, Bangladesh has achieved one of the world's most remarkable economic growth rates, averaging 4.0% annually^7^. Bangladesh achieved an impressive decade of 7% GDP growth, exceeded the lower middle-income threshold in 2015, and effectively transitioned out of the least developed country (LDC) status^8^. Goldman Sachs Investment Bank has designated Bangladesh as one of the next 11 nations poised for rapid economic growth in the twenty-first century. In 2015, Bangladesh stood out with the second highest real GDP growth rate, reaching an impressive 6.4%^9^. However, despite its economic success, Bangladesh's traditional environmental achievements have generally not met expectations^10^. Carbon dioxide (CO_2_) emissions in 2019 were almost 0.6 metric tons lower per person in Bangladesh than they were in 2000^11^. It should be emphasized that the use of gaseous fossil fuels accounts for approximately two thirds of Bangladesh's national CO_2_ emissions (World Bank 2020), illustrating the country's predominant dependence on fossil fuels^5,12^. On the other hand, Bangladesh's overall greenhouse gas emissions have increased by more than 45% since 1990^13^. Air pollution resulting from greenhouse gas emissions is not the only source of environmental pollution in Bangladesh. For example, industrial effluents, household waste, and agricultural runoff are the main causes of Bangladesh's constant decline in water quality^14^. In addition, deforestation is a major cause of environmental pollution in Bangladesh due to the country’s susceptibility to natural disasters such as landslides and floods^15^. Therefore, addressing the causes of the degradation of Bangladesh's environmental quality has become a top priority on the government's policy agenda. With international obligations to formulate effective policies to reduce ${{\text{CO}}}_{2}$ and other greenhouse gas emissions, Bangladesh prioritizes transitioning to a low-carbon economy^16^. Bangladesh was one of the 196 countries and economies that ratified the Paris Agreement at the United Nations Climate Change Conference (COP21) in December 2015^3,17^. Bangladesh ranks seventh in the global climate risk index in 2021 due to climate change, although it contributes less than 0.48% of global emissions and its significant impact^18^. Bangladesh must continue its efforts to become carbon neutral, focusing on greening production and consumption processes for environmental sustainability and international obligations, as it is one of the most vulnerable countries^19^. Limiting CO_2_ emissions is a national priority due to the severe impact on domestic industries, particularly agriculture, resulting from climate change^20^.

Global concerns about energy security have led developing countries to adopt reliable, cost-effective, and clean energy irrigation technologies to ensure food security, reduce pollution and enhance climate benefits^21^. The transition to solar energy could replace 10% of conventional energy sources, preserve fossil fuel reserves and ensure sustainable water management in agriculture^21,22^. Solar technology is increasingly popular around the world to promote climate-friendly renewable energy in production^23^. It not only reduces the dependence of farmers on expensive energy sources, but also reduces carbon dioxide emissions, improving both crop quality and quantity, and minimizing water wastage^23,24^. The government of Bangladesh is actively promoting the adoption of advanced technologies to harmonize energy and water resources in search of a more sustainable food production system^23^. Despite its ever-growing energy needs, Bangladesh has been heavily dependent on indigenous and imported fossil fuels. Consequently, the combustion of fossil fuels now accounts for a significant part of the country's overall energy consumption^13,25^.

In previous research, the probit model was widely used to assess various aspects of household energy choices, such as cooking and lighting preferences^26,27^. Some studies used multivariate probit estimates to evaluate energy choices^28^, while others used logit models to analyse factors determining the adoption of solar energy technologies^29^. A study conducted in China concentrated on the impact of non-farm employment on the adoption of clean energy^30^. While several studies have investigated the prevalence of clean energy adoption and its determining factors in some LMICs, there is a noticeable lack of research shedding light on this aspect in Bangladesh. This study aims to address this gap by outlining its objectives.

Addressing the existing research gap, our study primarily delves into examining the prevalence of clean energy adoption and its determining factors. Additionally, we explore the variations in energy adoption patterns between urban and rural households in Bangladesh, assessing the socioeconomic disparities in clean energy adoption within these demographic segments.

While a previous study in Bangladesh focused on identifying factors influencing solar adoption and their impact on welfare, utilizing the linear probability model (LPM) and probit regression, its scope was limited to solar home systems (SHS) ^31^. Acknowledging the potential issues of endogeneity inherent in adoption decisions, we applied an extended probit regression to investigate these factors and applied a Blinder-Oaxaca decomposition analysis to elucidate the disparities between urban and rural areas. It's noteworthy that, to the best of our knowledge, our present study is the first in Bangladesh to employ this comprehensive methodology in exploring the dynamics of clean energy adoption. The findings will be valuable for the government in aligning national goals with international goals like SDG 7.A (affordable and clean energy) and enhancing clean energy research and technology in developing countries like Bangladesh.

## Materials and methods

### Sources of data

This study used data from the Bangladesh Multiple Indicator Cluster Survey (MICS) 2019, a cross-sectional survey specifically designed to collect information on crucial indicators associated with the Sustainable Development Goals (SDGs). The Bangladesh Bureau of Statistics (BBS) conducted this national representative survey with the financial support of UNICEF.

### Sample design and sample size

The data was collected through a two-stage stratified cluster sampling approach. In the first step, 3,220 samples were collected from 08 divisions, and in the second stage 20 sample were gathered. Finally, out of a total of 64,400 households in 08 divisions, 61,602 households were successfully interviewed. The collected data were weighted to ensure that the survey findings accurately represented the country. As a result, the final sample size for the Bangladesh survey was 61,242 households.

### Dependent variable

The study dependent variable is the adoption of clean energy. The household's decision regarding clean fuels is binary, presenting two mutually exclusive outcomes: the use of clean fuels or unclean fuels^32^. Those household were used elective stove, liquefied petroleum gas (LPG), piped natural gas stove and biogas stove for cooking were considered as clean fuel adopters’ categories otherwise it is considered non clean fuel adopters’ categories^30^.

$$\text{Adoption of clean energy (ACE)}=\left\{\begin{array}{ll}1,& If household use clean fuel technologies for cooking \\ 0, & If household did not use clean fuel technologies for cooking\end{array}\right.$$

### Independent variables

Along with the dependent variable, we also take into account a respondent's division (Barishal, Chattogram, Dhaka, Khulna, Mymensingh, Rajshahi, Rangpur and Sylhet), type of residence (urban, rural), sex of household head (male, female), household head education (No education, primary, secondary and above), ethnicity of the head of household (Bengali, others), size of the household (< 4, 4–5, 6 +), age of the household head (15–64,65 +), Internet ( yes, no), dwelling (own, rent, others), mobile ( yes, no) as potential factors for this study. The wealth index is a composite measure of a household's wealth, calculated using principal components analysis. The wealth index is derived from data on a household's ownership of assets like televisions and bicycles, housing construction materials, and water access and sanitation facilities. It ranks households based on their assets and final factor scores, dividing them into five quintiles^33^. The survey population is ranked according to their wealth score and divided into five quintiles, from lowest to highest. In 2019, the Bangladesh Wealth Index used 25 variables to construct the index, aiming to capture long-term wealth through household assets^33^. The index ranks households from poorest to richest, based on their wealth score. The poor category was formed by merging the poorest and poorer groups of study participants, while the rich category was created by combining the richest and richer groups. Wealth index can be classified as three categories (poor, middle and rich). Empirical studies show a positive correlation between income and clean fuel use^34^. Studies in Bangladesh ^35^, Bhutan^36^, and Pakistan^37^ also confirm a positive association between income and wealth and the use of clean fuels.

### Analysis procedure

In this study, the summary of explanatory factors was presented using a percentage frequency distribution. We used two-way contingency tables and chi-square tests to analyze the connection between independent and dependent variables, as well as the association between sociodemographic components. Mathematically, chi-square statistics can be defined as

1 $$\chi^{2} = \mathop \sum \limits_{i = 1}^{n} \frac{{({\text{observed frequency}}\; y_{i} - {\text{Expected frequency }}y_{i} )^{2} { }}}{{{\text{Expected frequency }}y_{i} }}$$

This metric is based on a chi-square distribution, with (Number of rows – 1) × (Number of columns – 1) degrees of freedom.

### Extended probit regression

A Probit Regression was conducted to assess how wealth quintile influences the choice of adopting clean energy within households.

The specific model is:

2 $${\text{ACEi}} = \alpha + \beta_{1} \;{\text{Wealth Index}}\;{ + }\beta_{{2}} C + \varepsilon$$

In Formula (2), the dependent variable ACEi is the dependent variable (binary variable) indicates whether the respondents use clean energy or not; the explanatory variable wealth index (it has been classified as poor, middle, and rich); $\varepsilon$ is the coefficient to be evaluated; C represents the control variables, including individual, household and community characteristics; $\varepsilon$ is the random disturbance term.

As the decision to adopt clean energy (which depends on self-decision) is assumed to be correlated with unobservable time-varying factors, endogeneity issues are more likely to be found. The study considers potential endogeneity issues that could lead to biased estimates when examining the effects of the wealth index on the adoption of clean energy. In order to address this concern, extended probit regression models (EPRMs) were used^30^. These models help to take into account for unobservable factors and improve the reliability of the study's estimates. These models can accommodate various endogenous covariates, including continuous, binary or ordinal covariates, using the maximum likelihood estimation.

### Inequality analysis

In this equation, CIX represents the concentration index, ${R}_{i}$ denotes the fractional rank in the distribution of socioeconomic position, ${M}_{i}$ refers to the dependent variable index, and $\overline{M }$ signifies the mean of the outcome variable within the sample.

The major purpose of this study is to calculate an approximate CIX value using the Lorenz curve, also known as a concentration curve. If there is a discrepancy between the concentration curve and the 45° line, then there is probably no connection between the two. The range of the CIX value spans from − 1 to + 1, with the sign indicating the direction of any association between the health variable and socioeconomic position.

### Blinder–Oaxaca decomposition method

Using the Blinder-Oaxaca decomposition method^38^, we analyzed the factors that contributed to the average gap in clean energy adoption between urban and rural areas. Based on the formula in Eq. (2), the following regression model was developed.

3 $$\Delta \overline{Y }=\left({\delta }_{0}^{u}- {\delta }_{0}^{r}\right)+ \sum_{i=1}^{k}({\delta }_{i}^{u}{\overline{a} }_{i}^{u}- {\delta }_{i}^{r}{\overline{a} }_{i}^{r})$$

where, $\overline{a }$ represents the average of each predictor (covariate); *δ* represents the predicted regression coefficient; ‘u’ represents ‘urban group’; ‘r’ represents ‘rural group’; ($\Delta \overline{Y }$) represents predicted mean difference in clean energy adoption status between urban and rural groups.

The Blinder-Oaxaca decomposition method is a different approach, in which the coefficients and variable levels of one group are swapped with the corresponding values from another (reference group). We used the urban sample to determine what would happen to our projected mean when the rural sample was given the urban sample's values for the predictor variables and its regression coefficients. Decomposition models were defined using the formula in Eq. (3),

$$\Delta \overline{Y }=\left({\delta }_{0}^{u}- {\delta }_{0}^{r}\right)+ \sum_{i=1}^{k}{\delta }_{i}^{r}({\overline{a} }_{i}^{u}- {\overline{a} }_{i}^{r})+ \sum_{i=1}^{k}{\overline{a} }_{i}^{r}\left({\delta }_{0}^{u}- {\delta }_{0}^{r}\right)+ \sum_{i=1}^{k} \left({\delta }_{0}^{u}- {\delta }_{0}^{r}\right)\left({\overline{a} }_{i}^{u}- {\overline{a} }_{i}^{r}\right) (4)$$

Equation (4) is a decomposition model constructed from the perspective of the rural group, using the urban group as a point of comparison. Here, the expected mean difference ($\Delta \overline{Y }$) of clean energy usage status consists of four components, as given on the right-hand side of the equation.

- The first component revealed the influence of hidden characteristics.
- The second component revealed shifts in the average anticipated value of the rural group as it reached the factors' level of the urban group. It revealed how much variation in the level of the independent variables across groups may account for in the predicted mean difference ($\Delta \overline{Y }$). This section is called the "explained component" or the “endowments effect” in the literature.
- The third component reflected shifts in the average projected value for the rural group after they received the urban group's regression coefficients. It comprised the percentage of the predicted mean difference ($\Delta \overline{Y }$) attributable to the effect of the covariate on the result that varied between the urban and rural groups. This portion is termed the “unexplained component” or the “coefficient effect” in the academic literature.
- The fourth component resulted from the interaction between the effects of the differences in the endowments and the coefficients.

The three-fold decomposition model was made by combining the first component (i) which dealt with differences between two groups that could not be explained by the covariates included in the model, and the third component (iii), which also dealt with an unexplained portion of the difference, as shown in Eq. (4), to create the three-fold decomposition model^38^,

5 $$\Delta \overline{Y} = \mathop \sum \limits_{i = 1}^{k} \delta_{i}^{r} \left( {\overline{a}_{i}^{u} - \overline{a}_{i}^{r} } \right) + \mathop \sum \limits_{i = 1}^{k} \overline{a}_{i}^{r} \left( {\delta_{0}^{u} - \delta_{0}^{r} } \right) + \mathop \sum \limits_{i = 1}^{k} \left( {\delta_{0}^{u} - \delta_{0}^{r} } \right) \left( {\overline{a}_{i}^{u} - \overline{a}_{i}^{r} } \right)$$

The first, second, and third components on the right side of the equation, respectively, reflected the endowments effect, the coefficients effect, and the interaction effect.

A holistic decomposition was performed to determine the relative contributions of each independent variable to endowments, coefficients, and interaction. This involved gradually switching out one set of levels or coefficients for another set while keeping all other variables constant in the equation. The first, second, and third components on the right side of the equation, respectively, reflect the endowment effect, the coefficient effect, and the interaction effect. The Statistical Package for Social Science (SPSS) version 25.0 (IBM Corporation, Armonk, New York, New York, USA) was used for data administration. In this study, STATA version 15 was used for data analysis, and R software (version 4.0.0), along with the ggplot2 package, was used to generate a map.

## Ethical approval

Multiple Indicator Cluster Survey (MICS) data from three different areas were utilized for the analysis; this data is freely available at the following link: https://mics.unicef.org/. Since the research was conducted using publicly accessible secondary data, no further ethical approval was required for this work.

## Results

Table 1 presents the distribution of the participants in this study, highlighting key demographic and socioeconomic characteristics. The findings reveal that the highest percentage of participants came from the Dhaka division (25.3%), while the lowest proportion came from the Barishal division of Bangladesh. Most of the participants resided in rural areas (77.9%) and the heads of household consisted predominantly of males (87.3%).

**Table 1:** Background characteristics of study participants.

Variables	Frequency	Percentage
Individual Level Factor		
Division		
Barishal	3488	5.7
Chattogram	10,736	17.5
Dhaka	15,512	25.3
Khulna	7290	11.9
Mymensingh	4561	7.4
Rajshahi	8745	14.3
Rangpur	7229	11.8
Sylhet	3681	6.0
Residence		
Urban	13,564	22.1
Rural	47,678	77.9
Sex of household head		
Male	53,460	87.3
Female	7782	12.7
Household head education		
No education	21,459	35.0
Primary	16,587	27.1
Secondary and above	23,196	37.9
Religion		
Muslim	55,261	90.2
Non-Muslim	5981	9.8
Ethnicity of household head		
Bengali	60,527	98.8
Others	715	1.2
Wealth index		
Poor	25,373	41.4
Middle	11,895	19.4
Rich	23,974	39.1
Household size		
< 4	20,894	34.1
4–5	28,758	47.0
6 +	11,590	18.9
Age		
15–64	53,243	86.9
65 +	7999	13.1
Internet		
Yes	23,013	37.6
No	38,229	62.4
Dwelling		
Own	51,458	84.0
Rent	7968	13.0
Others	1816	3.0
Mobile		
Yes	58,054	94.8
No	3188	5.2
Clean energy adopter		
Yes	12,210	19.9
No	49,032	80.1

Furthermore, the information in Table 1 shows that a higher proportion of household heads had completed secondary education or higher (37.9%) and the majority identified themselves as Muslim (90.2%). The ethnicity of the head of the household is also considered, highlighting the number and percentage of participants classified as Bengali (98.8%) or belonging to other ethnicities (1.2%).

Socioeconomic status is assessed using the wealth index variable, classifying study participants as poor (41.4%), middle (19.4%), and rich (39.1%). In terms of household size, the majority consisted of 4–5 members (47%), and a large portion of household heads feel within the age range of 15–64 (86.9%). In terms of Internet use, the majority did not use the Internet (62.4%), but almost all household heads owned a mobile phone (94.8%). Furthermore, most of the participants had their own dwellings (84%). Approximately one fifth of households used clean energy sources (19.9%), while the remaining 80.1% relied on other energy sources.

**Figure 1:**
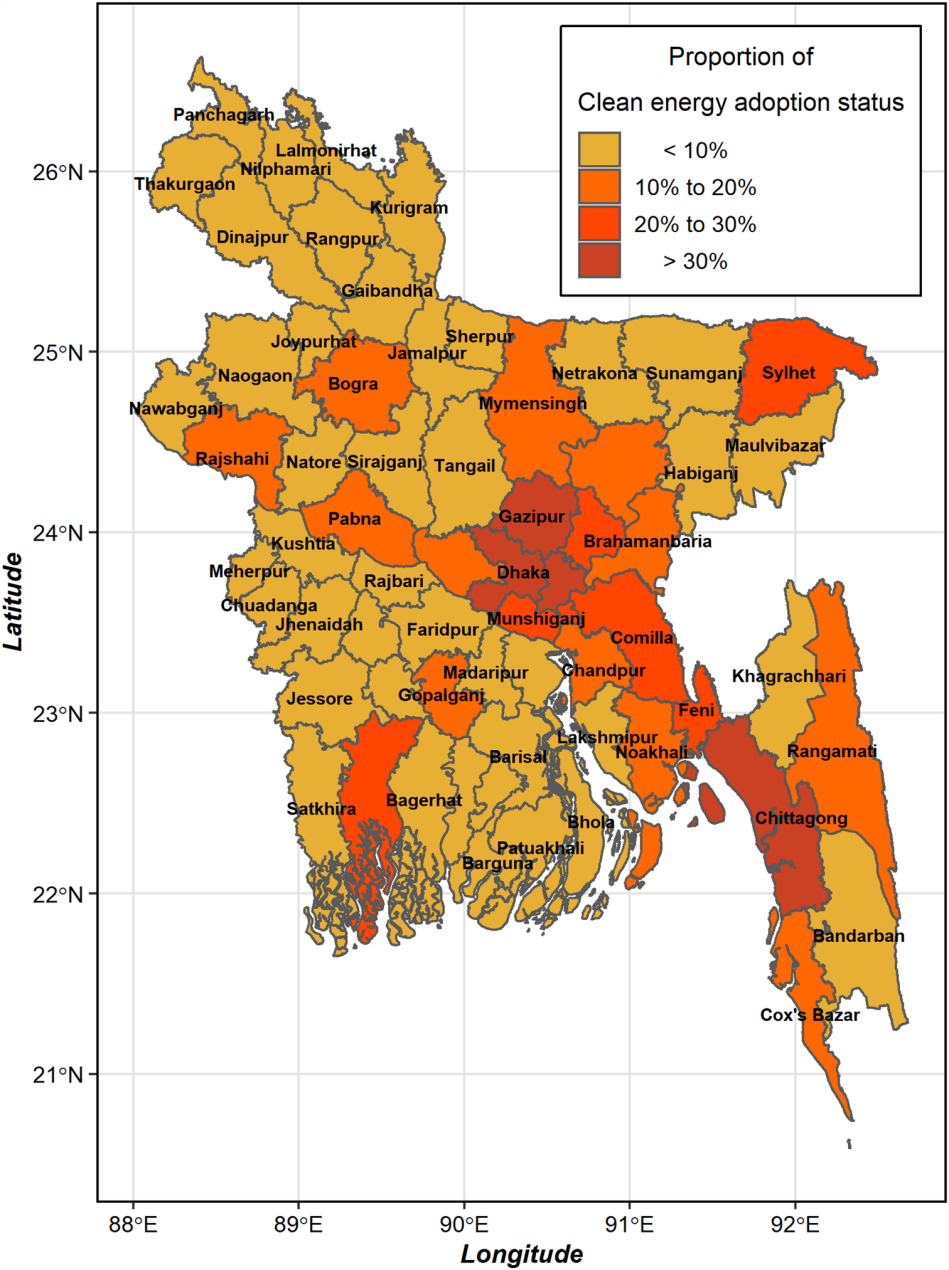
District wise household clean energy adoption status in Bangladesh.

Figure 1 shows the district-wise decision of the household to choose the adoption of clean energy. The district of Dhaka and Narayongonj showed the highest levels of clean energy adoption, while the district of Lalmonirhat and Kurigram showed the lowest levels of clean energy adoption.

Table 2 summarizes the sociodemographic characteristics of Bangladesh and the prevalence of households using clean energy to cook. The table highlights significant associations between the adoption of clean energy and various factors, including division, residence, sex of the household head, education of the household head, religion, ethnicity, wealth index, household size, age of the household head, mobile ownership, Internet access, and dwelling type (*p* < 0.05).

**Table 2:** Association between cofactors and use of clean energy in Bangladesh.

Variables	Clean energy		χ^2^ Value (*p*-value)
	Non adopter (%)	Adopter (%)	
Division			
Barishal	95.50%	4.50%	9224.152 (< 0.001)
Chattogram	76.20%	23.80%	
Dhaka	55.60%	44.40%	
Khulna	91.90%	8.10%	
Mymensingh	91.50%	8.50%	
Rajshahi	91.30%	8.70%	
Rangpur	95.00%	5.00%	
Sylhet	86.10%	13.90%	
Residence			
Urban	40.50%	59.50%	17,086.867 (< 0.001)
Rural	91.30%	8.70%	
Sex of household head			
Male	80.70%	19.30%	108.169 (< 0.001)
Female	75.70%	24.30%	
Household head education			
No education	90.80%	9.20%	4249.15(< 0.001)
Primary	84.50%	15.50%	
Secondary and above	67.00%	33.00%	
Religion			
Muslim	79.30%	20.70%	181.668 (< 0.001)
Non-Muslim	86.70%	13.30%	
Ethnicity			
Bengali	79.90%	20.10%	80.942 (< 0.001)
Others	93.40%	6.60%	
Wealth index			
Poor	99.80%	0.20%	21,338.894 (< 0.001)
Middle	97.20%	2.80%	
Rich	50.70%	49.30%	
Household Size			
< 4	76.10%	23.90%	456.836 (< 0.001)
5-Apr	80.60%	19.40%	
6 +	85.90%	14.10%	
Age			
15–64	78.90%	21.10%	325.962 (< 0.001)
65 +	87.60%	12.40%	
Internet			
Yes	67.00%	33.00%	3938.143 (< 0.001)
No	87.90%	12.10%	
Dwelling			
Own	89.30%	10.70%	21,946.994 (< 0.001)
Rent	18.20%	81.80%	
Others	90.80%	9.20%	
Mobile			
Yes	79.40%	20.60%	337.901 (< 0.001)
No	92.70%	7.30%	

The data show that households in the Dhaka division had the highest proportion (44.4%) of clean energy consumption for cooking, followed by households in the Chattogram division (23.8%). The adoption of clean energy was significantly higher in urban households (59.5%) compared to rural households (8.7%). In addition, households with female heads (24.3%) used clean energy slightly more than those with male heads (19.3%).

Regarding education of household heads, those household heads who had completed at least secondary education showed a significantly higher use of clean energy (33%). Muslim households were more likely to adopt clean energy (20.7%) than non-Muslim households (13.3%). Additionally, households belonging to the Bengali ethnic group had a significantly higher proportion of clean energy use (20.1%) than households belonging to other ethnic groups.

The wealth index was also an important factor, since a significant proportion of rich households (49.3%) use clean energy, compared to only a small percentage of middle-class households (2.8%). The size of households showed an inverse relationship with the use of clean energy, households with less than four members showing a higher percentage (23.9%) compared to households with four to five members and those with more than five members. Mobile ownership was also associated with higher clean energy use (20.6%) compared to households without mobile phones. Similarly, households with Internet access showed a significantly higher percentage of clean energy use (33%). Furthermore, the type of dwelling played a role, as households living in rented dwellings showed the highest percentage of clean energy use (81.8%).

### Factors associated with clean energy adoption in Bangladesh

The parameter estimates of the extended probit regression model are shown in Table 3. In this study, we assessed multicollinearity using the variance inflation factor (VIF) and the tolerance limit (TL). Multicollinearity is usually identified when the variance inflation factor (VIF) exceeds a threshold of 5 or 10^21,39^, or when the tolerance limit falls below 0.1 or 0.2^39^. Specifically, the calculated VIF values ranged from 1.054 to 2.506, which is well below the conventional threshold of 5 and TL > 0.2. This indicates that multicollinearity was not an issue in our analysis.

**Table 3:** Extended probit model estimates of household clean energy adoption.

Variables	Clean energy adoption			Wealth Index
	Poor (coefficient)	Middle (coefficient)	Rich (coefficient)	Coefficient
Wealth index				
Poor	− 1.724***			
Middle	− 2.363***			
Rich	− 2.809***			
Age				
15–64 versus. 65 + (ref)	− 0.136*	− 0.118**	− 0.047	− 0.209***
Religion				
Muslim versus Non-Muslim (ref.)	− 0.1	0.122*	0.220***	
Household size				
< 4 versus 6 + (ref.)	− 0.182**	0.104*	0.347***	− 0.073***
4–5 versus 6 + (ref.)	− 0.153**	− 0.042	0.169***	− 0.071***
Household head education				
No education versus Secondary and above (ref.)	− 0.741***	− 0.706***	− 0.762***	− 0. 885***
Primary versus. Secondary and above (ref.)	− 0.500***	− 0.520***	− 0.509***	− 0.615***
Sex of household head				
Male versus Female (ref.)	− 0.157*	0.093*	0.009	
Ethnicity				
Bengali versus Others(ref)	0.761***	0.663***	0.415***	0.992***
Dwelling				
Own versus Others(ref)	0.432**	0.253**	0.184**	0.478***
Rent versus Others(ref)	1.300***	1.475***	1.630***	1.494***
Mobile				
Yes versus No(ref)	0.402***	0.435***	0.318***	0.565***
Internet				
Yes versus No(ref)	0.800***	0.641***	0.591***	0.972***
Residence				
Urban versus Rural (ref.)	0.613***	0.609***	1.084***	0.776***
Division				
Barishal versus Sylhet (ref.)	− 0.681***	− 0.783***	− 0.954***	− 0.815***
Chattogram versus Sylhet (ref.)	− 0.008	0.036	0.188***	− 0.047
Dhaka versus Sylhet (ref.)	− 0.006	0.068	0.421***	0.074**
Khulna versus Sylhet (ref.)	− 0.384**	− 0.315***	− 0.516***	− 0.088***
Mymensingh versus Sylhet (ref.)	− 0.451***	− 0.495***	− 0.285***	− 0.464***
Rajshahi versus Sylhet (ref.)	− 0.302**	− 0.388***	− 0.383***	− 0.189***
Rangpur versus Sylhet (ref.)	− 0.598***	− 0.602***	− 0.459***	− 0.517***
Correlation between wealth index and clean energy adoption	0.883***			

Number of observations: 61,242

Wald chi^2^: 28,133.37***

Log pseudolikelihood: − 59,393.53

ref. = Reference Category; Statistical Significance: *p < 0.05; **p < 0.01; *** *p* < 0.001.

The likelihood ratio test (Wald chi-squared) of the overall model was highly significant (*p* < 0.001), indicating that the model has a strong explanation power. In order to examine the endogeneity of wealth status and the adoption of clean energy, we analyzed the correlations between the error terms in the equations. We found that the correlation between the errors of our two equations was 0.883 and significantly different from zero (*p* < 0.001), indicating the presence of endogeneity. Moreover, because the correlation is positive, we can infer that unobservable factors that lead to an increase in wealth quintile also tend to increase the probability of adopting clean energy.

This effect shows that when the age of the head of household increases from one age group (15–64) to the next (65 +), the probability of adopting clean energy decreases by 13.6% and 11.8% in poor and middle households, respectively. Muslim households from middle and wealthy families exhibited a positive and statistically significant influence on household energy decisions toward cleaner fuels (*p* < 0.05). Muslim households are likelier to adopt cleaner fuels than their counterparts.

The study found that households heads without education and primary education had a negative impact on household adoption of clean energy compared to those with secondary and higher education (*p* < 0.001). For example, the transition from lack of education to achieving secondary or higher education is associated with an increase of 76.2% in the likelihood of choosing clean cooking fuels within the rich wealth quintiles. The study found that the size of the household, particularly those with less than four members, has a negative and statistically significant effect (*p* < 0.01) on the probability that poor households choose clean energy, while it also has significant positive effects on the probability that middle and rich households choose clean energy compared to households with more than six members. This means that when a household moves from having less than four members to having six or more members, the probability of adopting clean energy increases by 34.7%. For the gender of the household head, a negative coefficient suggests that if the household head changes from male to female, the probability of adopting clean energy decreases by a factor of − 0.157, which means a decrease in the likelihood. In terms of ethnicity, people speak the Bengali language were likelier to adopt cleaner fuels than their counterparts.

Renter households showed a higher preference for clean energy use compared to other types of housing (*p* < 0.001). Households with mobile phones were positively associated with the adoption of clean energy in Bangladesh. This effect implies that when switching from not having a mobile phone (no category) to having one (yes category), the likelihood of selecting clean cooking fuels increases by 40.2%, 43.5% and 31.8% for poor, middle and rich households, respectively. Internet access significantly influenced households to adopt clean energy, showing a positive impact on energy consumption. Urban dwellers are likelier to adopt cleaner fuels than their counterparts. This effect suggests that the transition from rural to urban areas led to an increase of 61.3% in the likelihood of selecting clean cooking fuels within the poor wealth quintiles. Similarly, in the rich wealth quintiles, the probability of opting for clean cooking fuels increased by 108.4% when moving from rural to urban areas. Unlike the wealthy category, both the poor and middle wealth quintile categories demonstrated a substantial and negative impact on the adoption of clean energy sources by households in the Barisal, Khulna, Mymensingh, Rajshahi and Rangpur divisions compared to the Sylhet division (*p* < 0.05). However, it is important to note that households in the rich quintile of the Chattogram and Dhaka divisions showed a significantly positive impact on the adoption of clean energy compared to the Sylhet division. However, this effect was statistically not significant in poor and middle-class households (*p* > 0.05).

### Socioeconomic inequalities of urban and rural groups clean energy adoption

**Figure 2:**
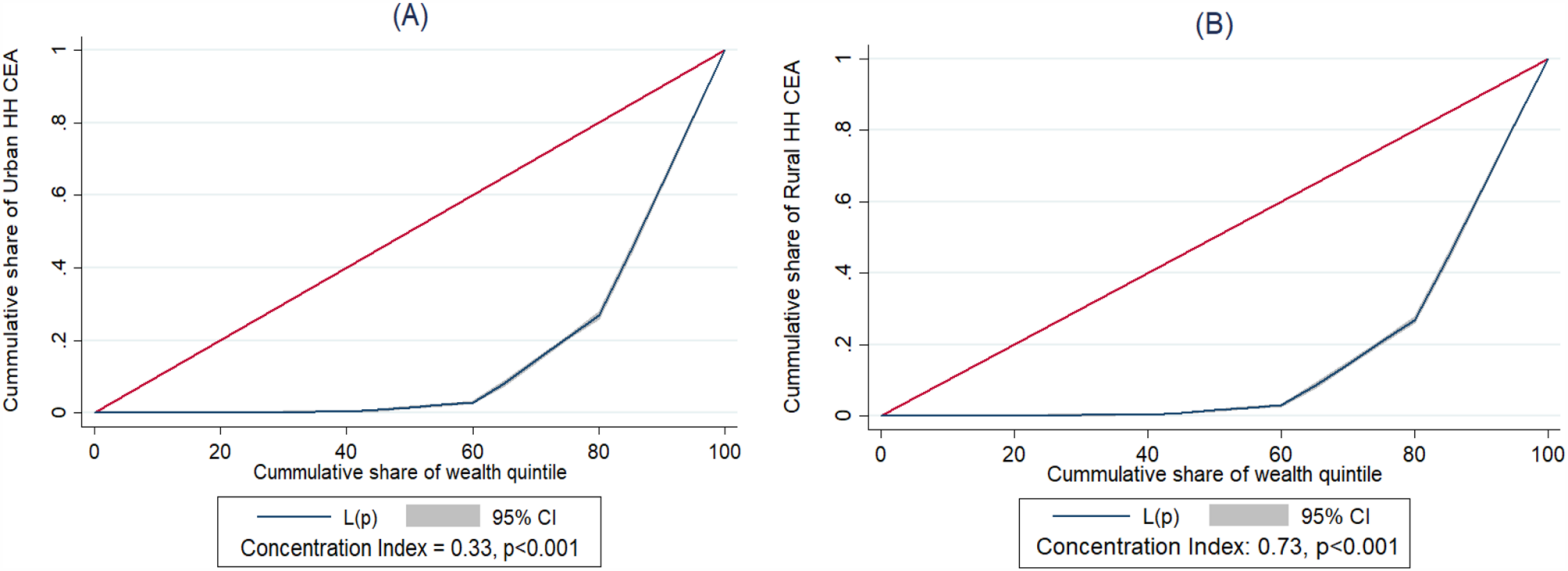
Socioeconomic inequalities of urban (**A**) and rural (**B**) groups clean energy adoption in Bangladesh.

Figure 2 shows the results of the concentration index used to assess the socioeconomic disparities in the adoption of clean energy between urban and rural groups in Bangladesh. The results indicate that about 33% of the socioeconomic inequality is observed in the adoption of clean energy in urban groups (CI = 0.33). In Fig. 2A, the concentration curve is located below the diagonal line, indicating that the disparities in the adoption of clean energy are more pronounced among the richest quintile in urban groups. Furthermore, approximately 73% of the socioeconomic inequality in the adoption of clean energy among rural groups has been identified (CI = 0.73). Figure 2B shows the concentration curve below the diagonal line, indicating that the disparities in the adoption of clean energy are more concentrated among the richest quintile within rural groups.

### Results from Blinder–Oaxaca decomposition

Table 4 shows the results of the Blinder-Oaxaca decomposition analysis conducted to investigate the patterns of adoption of clean energy that highlight the contributions of endowments, coefficients and interactions to explain the disparities in the adoption of clean energy between urban and rural areas in Bangladesh.

**Table 4:** Blinder-Oaxaca decomposition estimates of clean energy adoption status in Bangladesh.

	Co-efficient	Standard error	*p*-value	95% CI
Predicted probability				
Urban	0.595***	0.005	< 0.001	(0.586, 0.604)
Rural	0.087***	0.001	< 0.001	(0.084, 0.089)
Difference in predicted probability				
Total difference (R)	0.508***	0.005	< 0.001	(0.499, 0.517)
Decomposition				
Difference due to endowments (E)	0.408***	0.004	< 0.001	(0.400, 0.415)
Difference due to coefficients (C)	0.178***	0.005	< 0.001	(0.168, 0.189)
Difference due to interaction (CE)	− 0.078***	0.004	< 0.001	(− 0.086, − 0.069)

In Bangladesh, the likelihood of adopting clean energy was about 60% (0.595) among urban residents and about 9% (0.087) among rural residents. This implies that urban residents are more likely to adopt sustainable energy than their rural counterparts. The average general difference between urban and rural areas was 0.508 (95% CI 0.499 to 0.517, *p* < 0.001).

This difference is divided into three components: endowments, coefficients, and interactions. The disparity between urban and rural areas attributed to endowments (E) accounted for approximately 80.31% (0.408) (95% CI 0.400 to 0.415, *p* < 0.001) of the observed variation. This indicates that discrepancies in the characteristics or attributes of the respondents contribute to about 80.31% of the disparities between urban and rural areas of Bangladesh.

Furthermore, the disparity between urban and rural areas due to coefficients (C) was approximately 35.04% (0.178) (95% CI 0.168 to 0.189, *p* < 0.001) of the observed variation. This suggests that differences in the relationships between the characteristics of the respondents and the adoption of clean energy account for approximately 35.04% of the disparities between urban and rural areas in Bangladesh.

Finally, the disparity between urban and rural areas resulting from interactions (CE) was approximately − 15.35% (− 0.078) (95% CI − 0.086 to − 0.069, *p* < 0.001). This indicates that differences in interaction or combinations of the characteristics of the respondents play a significant role, accounting for approximately − 15.35% of the variances between urban and rural locations in Bangladesh.

The complete results of the Blinder-Oaxaca decomposition study are shown in Table 5, which illustrates the roles played by several groups of variables in the establishment of the endowment effect and the coefficient effect and the explanation of the clean energy disparities between urban and rural households in Bangladesh. The absolute and relative importance of each variable and relative importance of each category within this variable are provided by the results. This in-depth investigation has been useful in identifying the types of variables that have the greatest impact on the different aspects of the gap.

**Table 5:** Blinder-Oaxaca decomposition showing contribution of all categories of variables towards endowments effect, and coefficients effect, Bangladesh MICS 2019.

Variables	Endowments contribution (%)	*p*-value	Coefficient contribution (%)	*p*-value
Wealth index				
Poor	55.440***	< 0.001	− 20.741**	0.006
Middle	9.892***	< 0.001	− 13.940***	< 0.001
Rich (ref.)				
Age				
15–64	− 0.017	0.87	− 15.46	0.145
65 + (ref)				
Religion				
Muslim	0.147**	0.005	7.081	0.561
Non-Muslim (ref.)				
Household size				
< 4	0.345***	< 0.001	− 10.797**	0.008
4–5	0.052	0.327	− 3.86	0.419
6 + (ref.)				
Household head education				
No education	4.237***	< 0.001	− 14.896***	< 0.001
Primary	0.972***	< 0.001	− 12.244***	< 0.001
Secondary and above (ref.)				
Sex of household head				
Male	0.001	0.898	− 21.273*	0.033
Female (ref.)				
Ethnicity				
Bengali	0.049	0.088	148.457***	< 0.001
Others(ref)				
Dwelling				
Own	− 0.517	0.802	18.899	0.2
Rent	17.742***	< 0.001	− 14.997	0.217
Others(ref)				
Mobile				
Yes	− 0.118	0.43	− 18.421	0.487
No(ref)				
Internet				
Yes	2.001***	< 0.001	20.421**	0.001
No(ref)				
Division				
Barishal	0.851***	< 0.001	− 2.768**	0.003
Chattogram	0.274**	0.008	7.033*	0.018
Dhaka	5.584***	< 0.001	12.938*	0.042
Khulna	0.993***	< 0.001	− 2.384	0.136
Mymensingh	0.142	0.368	− 0.198	0.847
Rajshahi	0.492**	0.002	2.447	0.208
Rangpur	1.360***	< 0.001	− 2.777*	0.033
Sylhet (ref.)				

Significant at ***p < 0.001; **p < 0.01; *p < 0.05.

In terms of the effect of endowments, the largest contribution maximized the urban–rural gap of the poor households category, accounting for 55.440% of the overall effect (*p* < 0.001). It was followed by the variables dwelling ownership type rent (17.742%), middle wealth quintile household (9.892%), Dhaka division (5.584%), household head with no education (4.237%), internet exposure (2.001%), Rangpur (1.360%), Khulna (0.993%), household head with primary education (0.972%), Barishal (0.851%), Rajhsahi (0.492%) and household size less than four (0.345%). These factors have played an important role in contributing to the overall endowment effect and were the most important contributors to explaining the gap between urban and rural residences.

Similarly, with regard to the effect of the coefficients, the greatest contribution came from the ethnicity of the head of Bengali , constituting a significant 148.457% of the effect (*p* < 0.001). It was followed by internet exposure (20.421%), Dhaka division (12.938%), and Chattogram division (7.033%). These factors have exerted considerable influence on shaping the coefficient effect. Factors such as male household head (− 21.273%), poor household (− 20.741%) household head with no education (− 14.896%), middle class household (-13.940%), household head with primary education (− 12.244%), household with fewer than four adults (− 10.797%), and Rangpur division (− 2.777%) and Barishal division (− 2.768%) have a more protective effect on the adoption of clean energy in the urban–rural gap.

## Discussion

Using data from the 2019 multiple indicator cluster surveys in Bangladesh, we tried to assess what variables could explain the growing popularity of renewable energy sources in Bangladesh. The results show that only 20% of households use renewable energy sources, while 80% use conventional sources. Biomass accounts for 68% of Kenya's primary energy use and is used by almost three-quarters of the population for basic energy needs^40^.

This study revealed that urban households are likelier to adopt cleaner fuels than their counterparts. It is similar to previous studies conducted in Cameroon^41^. In developing countries, impoverished urban households rely on solid fuels for cooking due to insufficient supplies, high costs of clean fuels and limited access to clean energy, as highlighted by various studies^34,35,42^.

The level of education of household heads also demonstrated a significant and positive relationship with the use of clean energy. On the contrary, households with lower educational levels were less likely to adopt cleaner fuels than their counterparts. This is because a cleaner, more sustainable lifestyle can help the environment in many ways. Previous studies^29,43^ found that household head education had a significant positive impact on decision-making on the adoption of biogas and solar energy. Previous studies have shown a positive relationship between educational levels and the adoption of clean energy sources, while negative associations are observed with the use of polluting fuels^44–46^. Furthermore, education level shows positive and significant correlations with the adoption of clean energy and a negative and significant association with the use of biomass and kerosene^28^. In particular, the educational background of the head of household is directly related to the adoption of clean energy and a reduced dependence on polluting fuels^47^. Education also has a favorable impact on the consumption of clean and non-traditional fuels, mainly due to the time-saving benefits they offer^35,42^. The probability of using clean fuels increases with higher education, whereas the probability of using polluting fuels decreases^44^. The study reveals a positive and statistically significant coefficient for the education of the household head on the preference for kerosene and natural gas as cooking fuels^48^.

Bengali families are leading in the adoption of clean energy. This study found that those ethnics who speak Bengali language are likelier to adopt cleaner fuels than their counterparts. In terms of rich wealth quintiles, this study found that households with a younger age between 15 and 64 were significantly less likely to use cleaner fuels than households with an older age of 65 and over. Contrary to our findings, a previous study showed that households with older heads were more likely to use cleaner fuels^49^. Baiyegunhi and Hassan (2014) observed that as households in rural Nigeria get older, they tend to switch from natural gas to wood fuel for cooking^48^. Younger farmers, particularly those under 30 years of age, show an increase of 0.27% in their willingness to adopt solar irrigation technology in Bangladesh^21^.

This study also found that Muslim households are likelier to adopt cleaner fuels than their counterparts. The size of the household was also significantly associated with the adoption of clean energy by households with positive and negative effects. For example, small households were less likely to adopt cleaner fuels than their counterparts in poor class household, while small households are likelier to adopt cleaner fuels than their counterparts in rich class household. The marginal effect analysis indicates that for each additional member added to the household, the likelihood of adopting clean cooking fuel increases by 2.4%^32^. Previous studies suggest that larger households tend to adopt energy-efficient practices more than smaller ones, which is consistent with previous findings^50,51^. Similarly, larger households prefer more efficient cooking methods due to reduced cooking time and the preservation of wood resources^48^. The impact of the size of the household on the adoption of solar PV can vary, with positive and negative effects^29^. On the one hand, larger households are more inclined to adopt solar PV energy due to their higher electricity consumption and the ability to distribute fixed costs^52^.

Interestingly, women in the poor headed household favored the decision to use clean energy than the poor male headed household. Female-led households prefer liquified petroleum gas (LPG) and electricity as cooking fuels, while reducing their use of kerosene and coal, a finding consistent with previous studies^28,34^. Regarding solar energy technology, male-headed households are more likely to resist its adoption compared to female-headed households^29^. Gender plays an important role in household energy decisions, and female-headed households prefer modern fuels^51^, contrary to previous studies in rural Nepal, which reported a preference for traditional fuels^53^.

There was a significant and positive association between the wealth index and the adoption of clean energy. For example, households in the poor category were less likely to adopt cleaner fuels than their counterparts. According to the results of this study, households with higher income levels demonstrate a greater probability of adopting cleaner cooking fuels^32^. A previous study had documented a positive correlation between rising household income and electricity consumption^54^. Guta (2018) has posited that an increase in household income enhances its capacity to cover expenses associated with solar energy, thus increasing the probability of its adoption^29^.

Furthermore, households living in rental homes were higher users of clean energy than those in other types of housing. The choice of cooking fuel for the home is influenced by various factors such as the age of the household head, family size, educational level, type of food preparation, fuelwood taste, and ownership of dwelling units^55^. Households residing in dwelling units are more inclined to use clean energy^30^. The study identified a positive and statistically significant coefficient of household ownership that demonstrated its substantial influence on the likelihood of switching from fuelwood to natural gas as the main cooking fuel^48^.

The home had a mobile phone that was positively correlated with the adoption of clean energy in Bangladesh. The study shows that households with Internet access are more likely to use clean energy than their counterparts. The impact of the use of the mobile Internet on the adoption of green technologies is significantly mediated by factors such as information acquisition capability, risk attitude, and expected return^56^. Econometric analysis shows that trade in ICT in South Asia has a positive impact on the energy sector by increasing renewable energy consumption, promoting renewable sources, reducing energy intensity, promoting cleaner cooking fuels and reducing carbon dioxide emissions^57^.

This study also found that households in the Dhaka and Chattogram divisions were more likely to use clean energy than in the Sylhet division. On the contrary, the Barisal, Khulna, Mymensingh, Rajshahi, and Rangpur divisions exhibited a significant negative effect on the adoption of cleaner energy sources in households compared to the Sylhet division. Although the richest quintiles have already switched to clean energy, there are still many people living in urban and rural areas who do not have access to cost-effective clean energy options. These individuals may find it difficult to take advantage of benefits associated with the adoption of clean energy, as a result of various types of inequity caused by this lack of access. The study also analyzed socioeconomic inequalities in the adoption of clean energy and found that these inequalities were more concentrated among wealthy quintiles in urban (33%) and rural (73%) areas of Bangladesh. Poor households often rely on solid fuels such as biomass, cow dung, and firewood and chips^58^. As income increases, they gradually switch to clean fuels such as LPG and electricity, according to the hypothesis of the energy ladder, according to various studies^37,59^.

We used the Blinder-Oaxaca approach to divide the adoption gap for clean energy into its components: endowments, coefficients, and interaction. Important contributors to each component have been isolated. Compared to urban and rural areas, the adoption rate of clean energy was 0.595 and 0.087, respectively. The difference in the adoption of clean energy between the groups was 0.508. Rural areas often trail behind in terms of the availability of renewable energy sources, leading to significant differences in the adoption of clean energy between urban and rural areas. Liquefied petroleum gas (LPG) is a feasible alternative for rural households. However, its substantial cost of refilling makes it financially inaccessible. This issue has led to the creation of improved cookstoves, designed to improve fuel efficiency and reduce emissions ^60^.

Many rural communities are unable to use these opportunities due to lack of resources, despite the potential advantages of clean energy, such as reducing environmental impacts and reducing energy bills. The average variation in the attributes of the study participants (endowments) accounted for 0.408 of the clean energy adoption gap. Equally important for the clean energy adoption gap were the disparities between groups in the impact of coefficients and interaction (0.178 and − 0.078, respectively).

The endowments covered the fraction of the clean energy adoption gap that could be effectively closed by raising public awareness in the rural population to reduce energy-related inequalities. Most of the impact of endowments was driven by the following: poor class household, rented home, middle class household, Dhaka, household head with no education, Internet exposure, Rangpur, Khulna, household head with primary education, Barishal division, Rajhsahi division, and household size (< 4). Given the significant group disparities, especially in the effects of predictors, it is not clear whether a policy intervention aimed at improving the level of predictors would be adequate to reduce the adoption gap in urban and rural areas. The main causes of the coefficients' influence were: male household head, poor household, household head with no education, middle class household, household head with primary education, household with fewer than four adults, Rangpur division and Barishal division had more protective effect on the adoption of clean energy in urban and rural gap. Therefore, other variables, such as providing some economic incentives, promoting awareness, and developing infrastructure, can play an essential role in reducing the gap in predictor impact. In addition, government policies and laws can help bridge the gap between populations.

The present study has both limitations and strengths. It did not assess significant variables like secondary income, economic growth^61^, energy use per capita^62^, political participation, and economic freedom^63^. Furthermore, this study relied on secondary data, making it difficult to establish a cause-and-effect relationship. Despite its limitations, this study has some strengths. The primary strength of this study was the rich database, which included nationally representative data with a substantial sample size of 61,242 households and provided valuable insights for policymakers and stakeholders in devising intervention strategies in rural areas of Bangladesh. This study used extended probit regression to investigate the factors and the Blinder-Oaxaca decomposition analysis to explain the disparities between urban and rural.

## Conclusion

This study identifies the potential factors associated with the adoption of clean energy by households in Bangladesh. To determine the factors that influence the adoption of renewable energy, extended probit regression was used. The factors influencing the gap in the adoption of clean energy were identified using the Blinder-Oaxaca decomposition method. Compared to rural areas, urban areas have a six fold increase in the adoption of clean energy. The current study shows that wealth index, religion, household size, household head education, household head sex, ethnicity, types of dwelling, mobile, Internet, residence and division were significant factors associated with the adoption of clean energy in Bangladesh households. The results showed that the richest quintile among rural groups had a greater focus on inequalities in the adoption of clean energy along the concentration curve. Unlike their counterparts in rural areas, urban dwellers were shown to be more likely to accept renewable energy based on the results of BO decomposition. According to the observed contribution, the category of poor households had the greatest impact on endowment. The Government of Bangladesh should be cautious in promoting the adoption of clean energy, particularly in rural areas. Policy makers can promote the adoption of clean energy options through media and online campaigns to raise awareness and training in rural communities, and to highlight the importance of clean energy. Additionally, efforts to increase education and reduce poverty in rural Bangladesh can contribute greatly to the successful adoption of clean energy options for rural people. The results of the study have an important impact on the government, policy makers, and other stakeholders in public health to increase the use of clean energy in households in Bangladesh through increased clean energy campaigns in rural areas of Khulna division, Barisal division, Rajshahi division, and Rangpur division to achieve the SDG.

## Data Availability

In this study, we used data from the 2019 Multiple Indicator Cluster Survey (MICS) in Bangladesh which is available from https://mics.unicef.org/surveys.
